# Mitochondrial m.1584A 12S m^6^_2_A rRNA methylation in families with m.1555A>G associated hearing loss

**DOI:** 10.1093/hmg/ddu518

**Published:** 2014-10-09

**Authors:** Mary O'Sullivan, Paul Rutland, Deirdre Lucas, Emma Ashton, Sebastian Hendricks, Shamima Rahman, Maria Bitner-Glindzicz

**Affiliations:** 1 Genetics and Genomic Medicine, UCL Institute of Child Health, London WC1N 1EH, UK,; 2 Nuffield Hearing and Speech Centre, Royal National Throat Nose and Ear Hospital, London WC1X 8DA, UK,; 3 NE Thames Regional Genetics Service,; 4 Metabolic Department, Great Ormond Street Hospital for Children NHS Trust, London WC1N 3JH, UK,; 5 Barnet and Chase Farm Hospitals NHS Trust, Enfield, Middlesex EN2 8JL, UK and; 6 Centre for Auditory Research, UCL Ear Institute, London WC1X 8EE, UK

## Abstract

The mitochondrial DNA mutation m.1555A>G predisposes to hearing loss following aminoglycoside antibiotic exposure in an idiosyncratic dose-independent manner. However, it may also cause maternally inherited hearing loss in the absence of aminoglycoside exposure or any other clinical features (non-syndromic hearing loss). Although m.1555A>G was identified as a cause of deafness more than twenty years ago, the pathogenic mechanism of this mutation of ribosomal RNA remains controversial. Different mechanistic concepts have been proposed. Most recently, evidence from cell lines and animal models suggested that patients with m.1555A>G may have more 12S rRNA N6, N6–dimethyladenosine (m^6^_2_A) methylation than controls, so-called ‘hypermethylation’. This has been implicated as a pathogenic mechanism of mitochondrial dysfunction but has yet to be validated in patients. 12S m^6^_2_A rRNA methylation, by the mitochondrial transcription factor 1 (TFB1M) enzyme, occurs at two successive nucleotides (m.1584A and m.1583A) in close proximity to m.1555A>G. We examined m^6^_2_A methylation in 14 patients with m.1555A>G, and controls, and found all detectable 12S rRNA transcripts to be methylated in both groups. Moreover, different RNA samples derived from the same patient (lymphocyte, fibroblast and lymphoblast) revealed that only transformed cells contained some unmethylated 12S rRNA transcripts, with all detectable 12S rRNA transcripts derived from primary samples m^6^_2_A-methylated. Our data indicate that TFB1M 12S m^6^_2_A rRNA hypermethylation is unlikely to be a pathogenic mechanism and may be an artefact of previous experimental models studied. We propose that RNA methylation studies in experimental models should be validated in primary clinical samples to ensure that they are applicable to the human situation.

## INTRODUCTION

**Figure 1.**
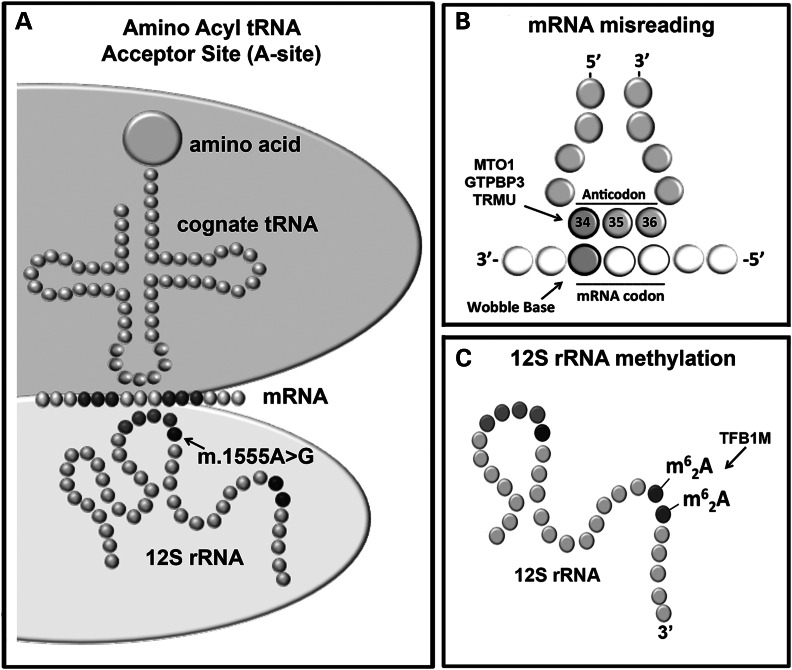
(**A**) m.1555A>G is located in the A-site, where the correct tRNA (cognate tRNA) anticodon and mRNA codon are matched during protein synthesis. (**B**) mRNA misreading has been postulated as a pathogenic mechanism for m.1555A>G mitochondrial dysfunction following aminoglycoside exposure, as the fidelity of translation decreases below a threshold level required for cochlear function. This mechanism is also consistent with the nuclear genes, *MTO1*, *GTPBP* and *TRMU*, reported to modify the penetrance of m.1555A>G hearing loss. (**C**) Evidence from transgenic cancerous cells and a transgenic mouse model indicates that patients with m.1555A>G may have more 12S m^6^_2_A rRNA methylation than controls, so-called hypermethylation. m^6^_2_A methylation occurs at two successive adenosines (m.1584A and m.1583A) on the 12S rRNA in close proximity to m.1555A>G.

One in 520 people carries a maternally inherited mitochondrial DNA (mtDNA) mutation, m.1555A>G, which predisposes to irreversible hearing loss following aminoglycoside antibiotic exposure (1,2). In a high proportion of cases, m.1555A>G leads to profound hearing loss following standard doses of aminoglycoside antibiotics (3). However, some family studies have reported maternally inherited deafness in the absence of aminoglycoside exposure. Because there are no additional clinical features, and to distinguish it from antibiotic-associated deafness, it has been termed ‘non-syndromic’ deafness (4,5). In these families, increased penetrance of m.1555A>G is attributed to nuclear modifier genes (5–8).

m.1555A>G is a homoplasmic mtDNA mutation, located in the *MT-RNR1* gene, which encodes the mitochondrial ribosomal 12S RNA (12S rRNA). The m.1555A>G 12S rRNA mutation is located in the ribosomal aminoacyl-tRNA-acceptor site (A-site), where the correct tRNA is matched to the correct mRNA codon during protein synthesis (Fig. 1A). The mitochondrial ribosome translates 11 mtDNA-encoded mRNAs into indispensable polypeptide subunits of the mitochondrial oxidative phosphorylation system (OXPHOS). OXPHOS is an essential biochemical energy production process involved in the synthesis of >90% of cellular ATP. The pathogenic mechanism of m.1555A>G is unclear, and two discordant mechanisms of mitochondrial dysfunction have been postulated (9–14). Broadly, these two mechanisms can be classified as *mRNA misreading* and *12S rRNA methylation* (Fig. 1B and C).

The first, long-standing hypothesis centres on the ability of the mitochondrial ribosome to discriminate between the correct, cognate tRNA and the incorrect, near-cognate tRNA during protein synthesis (Fig. 1B) (9,10). This hypothesis suggests that mitochondrial dysfunction in the presence of m.1555A>G occurs when the accuracy of ribosomal mRNA reading of 11 OXPHOS mRNAs has fallen below a minimum threshold level necessary for the basic metabolic requirements of cochlear cells (11). Early reports proposed this mechanism because it mirrors the orthologous mechanism of aminoglycoside action on gram-negative bacteria (4,9,10). Functional analysis of artificial hybrid ribosomes engineered to have m.1555A>G has since shown that m.1555A>G leads to a 7-fold increase in mRNA misreading and that mRNA misreading is further elevated in the presence of aminoglycoside antibiotics (11,15).

**Figure 2.**
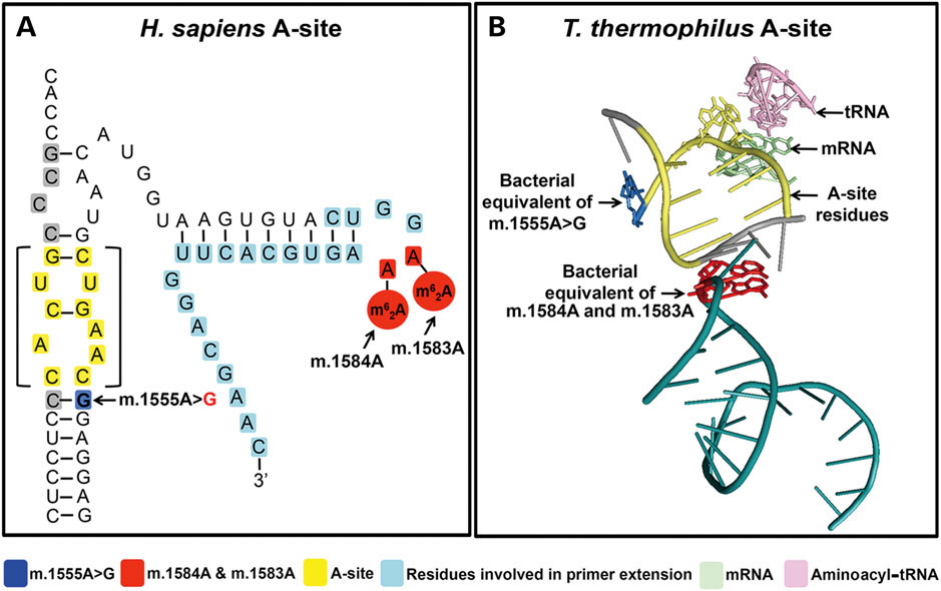
Evidence from experimental models proposes that m.1555A>G results in m.1584A and m.1583A m^6^_2_A hypermethylation - the proximity of these residues to m.1555A>G is shown in (**A**) the secondary structure of the 3′ end of the *H. sapiens* 12S rRNA and (**B**) the tertiary structure of *Thermo thermophilus* A-site (PDB: 3I8H).

In 2003, a second alternative hypothesis was proposed. Seidel-Rogul *et al.* hypothesized that the nuclear-encoded mitochondrial transcription factor 1 (TFB1M) enzyme may play a role in m.1555A>G hearing loss (Fig. 1C) (12). TFB1M belongs to a highly conserved family of RNA methyltransferases responsible for the N6, N6–dimethyladenosine (m^6^_2_A) methylation of two successive adenosine nucleotides near the 3′ end of small rRNA subunits in almost all species (16–18). In the *Homo sapiens* mitochondrial ribosome, the 12S rRNA is m^6^_2_A-methylated at positions m.1584A and m.1583A (Fig. 2A and B) (16,18). The highly conserved nature of the m^6^_2_A modification enzyme and the presence of two successive m^6^_2_A-methylated adenosines on almost all known small ribosomal subunits suggest a key cellular function for this modification (16–19). Studies indicate that m^6^_2_A methylation may act as a checkpoint during ribosome biogenesis, preventing immature small rRNAs from prematurely entering the translational cycle and causing ribosomal dysfunction (19). First reported in 1958, m^6^_2_A methylation is also known to modulate bacterial susceptibility to the aminoglycoside antibiotic kasugamycin, and studies have shown that, in a TFB1M knockout mouse, mitochondrial translation is severely impaired in the absence of m^6^_2_A rRNA methylation (12,19,20).

Seidel-Rogul *et al*. hypothesized that m^6^_2_A rRNA ‘hypermethylation’ on the mitochondrial 12S rRNA may influence the pathogenicity of m.1555A>G hearing loss (12–14). The term hypermethylation refers to an increase in the number of methylated residues in RNA or DNA compared with controls. The 12S rRNA hypermethylation hypothesis stems from the proximity of m.1583A and m.1584A to m.1555A>G (Fig. 2A and B) and the ability of m^6^_2_A methylation to modulate aminoglycoside susceptibility in bacteria.

Here, to establish the clinical relevance of 12S rRNA methylation in m.1555A>G hearing loss, 12S m^6^_2_A rRNA methylation was examined in 35 RNA samples - including primary lymphocyte RNA from 14 patients with m.1555A>G and 12 unrelated controls. A number of non-primary RNA samples from 143B.TK- and HeLa cell lines were also examined as controls.

## RESULTS

**Figure 3.**
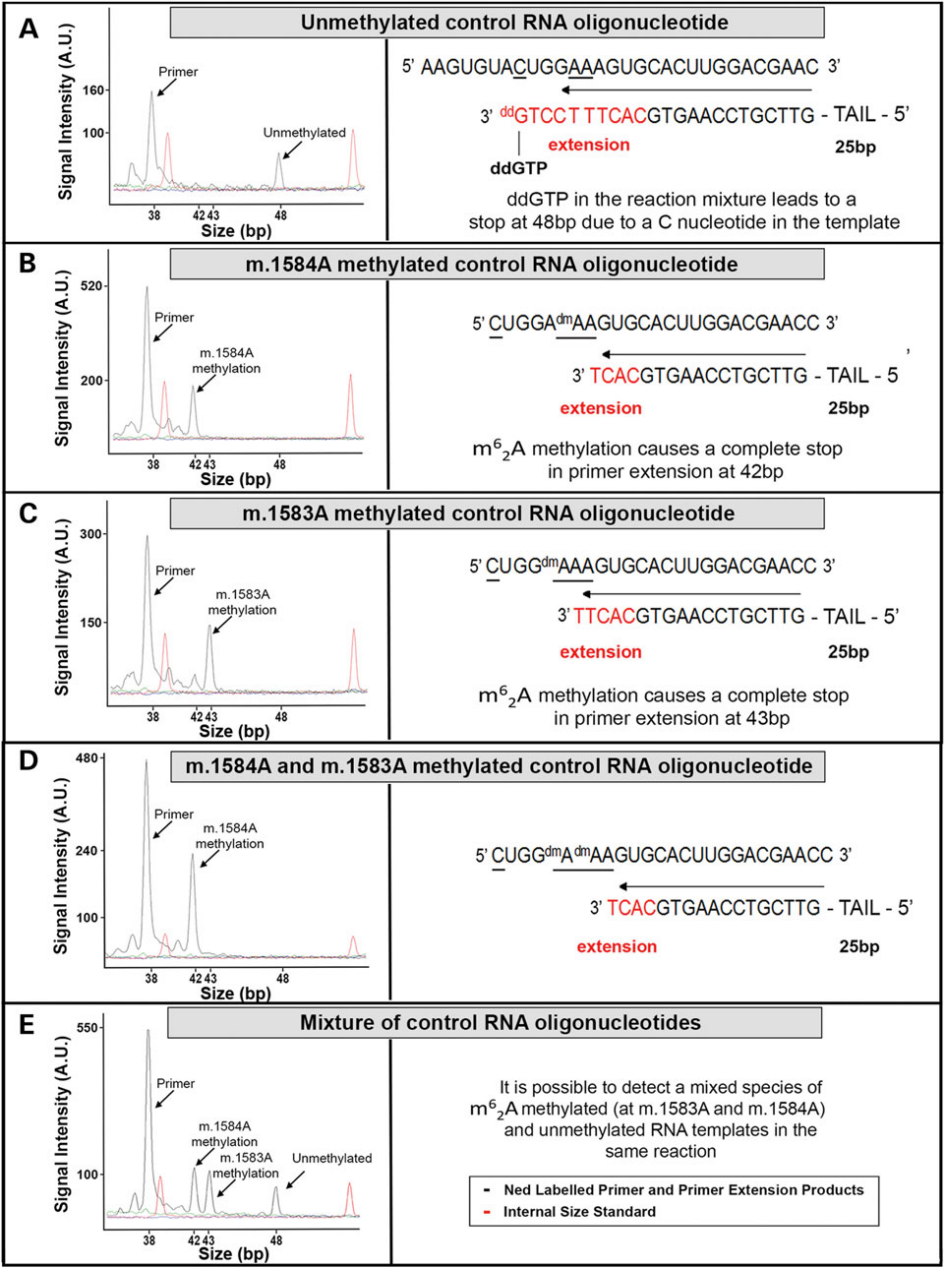
Primer extension of control synthetic RNA oligonucleotides, replicates of the reported native 12S rRNA methylated and unmethylated transcripts demonstrates: (**A**) termination of primer extension occurs after 48 bp when the RNA oligonucleotide is unmethylated, owing to the presence of ddGTP in the reaction and a cytosine nucleotide in the corresponding position in the template RNA; (**B**) an RNA oligonucleotide m^6^_2_A-methylated at the equivalent of m.1584A terminates at 42 bp, as the presence of m^6^_2_A methylation does not allow further extension; (**C**) m^6^_2_A methylation at the equivalent of m.1583A leads to termination at 43 bp; (**D**) primer extension on a template m^6^_2_A-methylated at the equivalent of m.1584A and m.1583A showed that it is not possible to detect m.1583A m^6^_2_A methylation in this situation. (**E**) All three templates present in the reaction together can be detected simultaneously in our primer extension assay. The primer is 38 bp in length.

To examine the potential role of TFB1M in m.1555A>G hearing loss, 12S m^6^_2_A rRNA methylation was examined using a fluorescent primer extension assay (Fig. 3) (21).

### Primer extension analysis of synthetic RNA templates

Four synthetic RNA oligonucleotides, designed to replicate the sequence of the mitochondrial 12S rRNA, were used as assay controls (Fig. 3). Analysis of synthetic unmethylated and methylated RNA oligonucleotides showed: (A) termination of primer extension occurs after 48 bp when the RNA oligonucleotide is unmethylated, owing to the presence of 2′,3′-Dideoxyguanosine-5′-Triphosphate (ddGTP) in the reaction and a cytosine nucleotide in the corresponding position in the template RNA; (B) an RNA oligonucleotide m^6^_2_A-methylated at the equivalent of m.1584A terminates at 42 bp, i.e. the presence of m^6^_2_A methylation does not allow further extension (21); (C) m^6^_2_A methylation at the equivalent of m.1583A leads to termination at 43 bp; (D) primer extension on a template m^6^_2_A-methylated at the equivalent of m.1584A *and* m.1583A showed that it is not possible to detect m.1583A m^6^_2_A methylation in this situation (Fig. 3). Figure 3 (E) shows that all three templates present in the reaction together can be detected simultaneously in our primer extension assay.

### Primer extension analysis of native RNA samples

**Figure 4.**
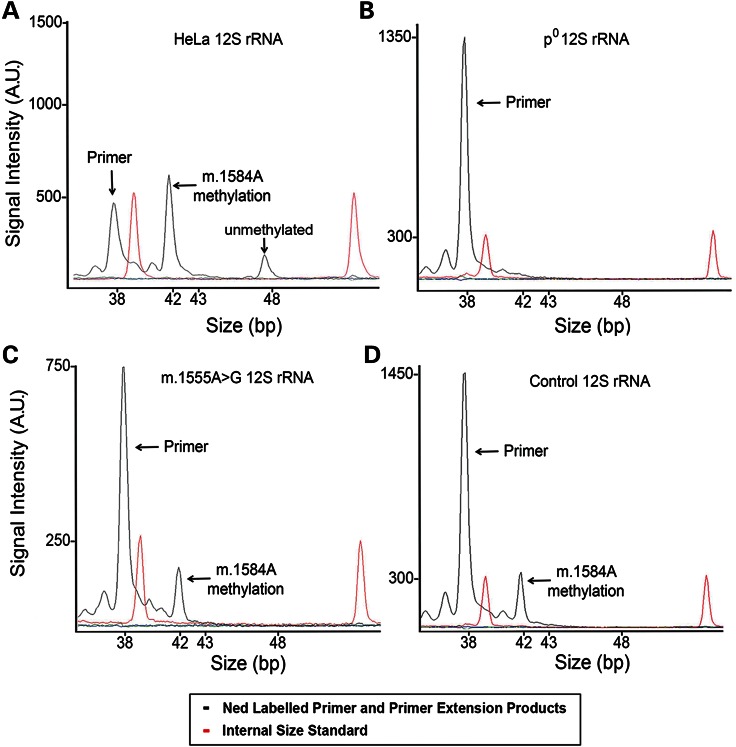
Primer extension of native RNA reveals: (**A**) HeLa 12S rRNA is partially methylated at m.1584A and unmethylated; (**B**) Control ρ° 206 RNA, containing no mtDNA, shows no primer extension; (**C**) all detectable m.1555A>G lymphocyte 12S rRNA appears to be fully methylated at m.1584A; (**D**) all detectable control lymphocyte 12S rRNA appears to be fully methylated at m.1584A.

Consistent with earlier findings by others, we found HeLa 12S rRNA and a 143B.TK− cell line to be partially methylated (Fig. 4A and Supplementary Material, Fig. S2A) (13). Mean percentage (±SD) of methylated HeLa 12S rRNA was 80.7 ± 0.3% (*n* = 3), calculated using area under the curve. Analysis of a control ρ^0^ 206 cell line, lacking mtDNA, showed no primer extension, confirming primer specificity to the mitochondrial transcriptome (Fig. 4B and Supplementary Material, Fig. S2).

**Figure 5.**
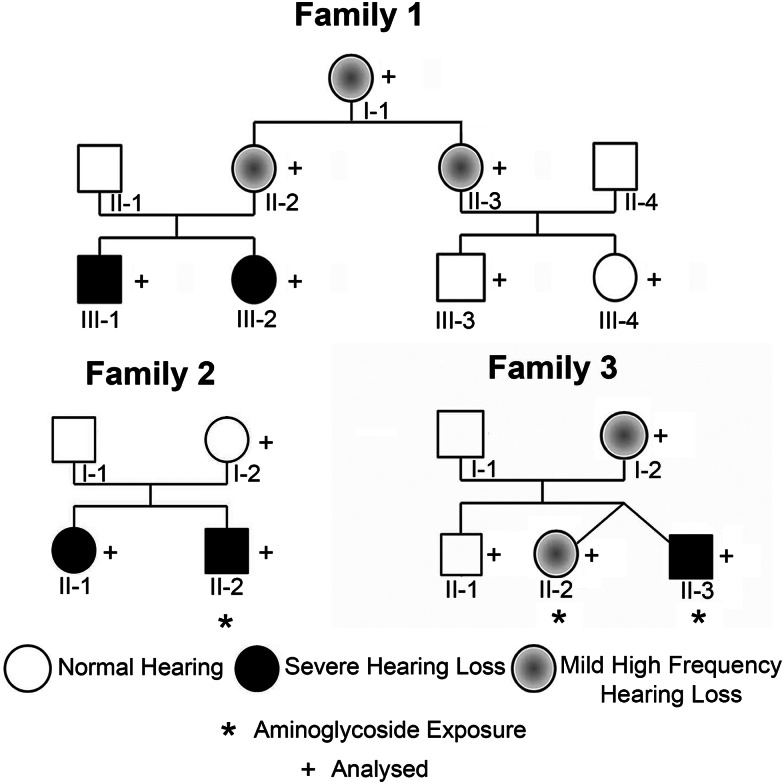
Primary lymphocyte 12S m^6^_2_A rRNA methylation was examined in 14 patients with m.1555A>G from three unrelated families. * Indicates aminoglycoside exposure. + Indicates individuals sampled.

Primer extension analysis of primary lymphocyte RNA from 14 patients with m.1555A>G (pedigrees shown in Fig. 5) and 12 controls showed that all detectable 12S rRNA appeared fully m^6^_2_A-methylated at m.1584A in both groups (Fig. 4 and Supplementary Material, Fig. S1). We found that the absolute quantity of 12S rRNA transcript in 10 µg of RNA varied between individuals, but in all cases, no unmethylated transcript could be detected using our fluorescent assay (Supplementary Material, Fig. S1). Individuals with mild hearing loss and severe hearing loss all showed full 12S m^6^_2_A rRNA methylation at position m.1584A with no detectable unmethylated transcripts.

**Figure 6.**
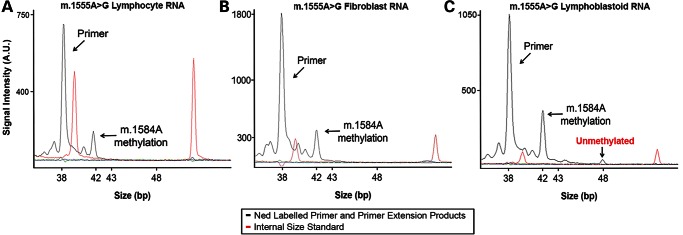
Primer extension analysis of 12S m^6^_2_A rRNA methylation in three different samples from the same patient (**A**) primary lymphocyte, (**B**) primary fibroblast and (**C**) transformed lymphoblastoid RNA. All detectable lymphocyte and fibroblast 12S rRNA appears to be fully methylated, but some 12S rRNA is unmethylated in the lymphoblastoid RNA.

Analysis of fibroblast, lymphocyte and lymphoblastoid cell-derived RNA from the same patient with m.1555A>G showed that 12S rRNA isolated from primary cells was methylated with no unmethylated species detected but, in marked contrast, some 12S rRNA isolated from the transformed lymphoblastoid cell line was unmethylated (Fig. 6). Unmethylated 12S rRNA was also observed in three further lymphoblastoid cell lines (Table 1). Thus, 12S m^6^_2_A rRNA methylation status appears to correlate with cell status (primary or not) but not the m.1555A>G genotype (Table 1).

**Table 1.** Summary of the primer extension results of the 35 native RNA samples analysed in this study

Species	Cell type	Cell status	Unmethylated 12S rRNA detected?	m.1555A>G	No. of samples	Replicates
*H. sapiens*	Lymphocyte	Primary	−	+	14	3^a^
*H. sapiens*	Fibroblast	Primary	−	+	1	3
*H. sapiens*	Lymphocyte	Primary	−	−	12	3
*H. sapiens*	Fibroblast	Primary	−	−	1	3
*H. sapiens*	Lymphoblastoid	Non-primary	+	+	2	2
*H. sapiens*	Lymphoblastoid	Non-primary	+	−	2	2
*H. sapiens*	HeLa	Non-primary	+	−	1	3
*H. sapiens*	143B.TK-	Non-primary	+	−	1	3
*H. sapiens*	143B.TK-ρ^0^	Non-primary	No primer extension	−	1	3
	Total number of samples analysed				35	

a Four patients were analysed in duplicate, as indicated in Supplementary Material, Figure S1.

## DISCUSSION

In 2003, Seidel-Rogul *et al*. hypothesized that 12S m^6^_2_A rRNA methylation may be important for mitochondrial dysfunction in the presence of m.1555A>G (12). The hypothesis stems from the proximity of m^6^_2_A-methylated nucleotides to m.1555A>G and the ability of m^6^_2_A methylation to modulate aminoglycoside susceptibility in bacteria *in vitro* (12). The first functional evidence suggesting TFB1M as a modulator of m.1555A>G hearing loss came from studies of transmitochondrial cybrids (13). These are transgenic cell lines containing the mitochondria from an immortal lymphoblastoid m.1555A>G cell line (itself derived from transforming peripheral blood lymphocytes) and the nucleus from a highly proliferative 143B.TK− osteosarcoma cancer cell line (13,22). Studies of this cybrid demonstrated that the 12S rRNA had relatively increased m^6^_2_A methylation at m.1584A and at m.1583A in a patient cell line (a 1 : 1 ratio of methylated : unmethylated transcripts) compared with a control cell line (a 1 : 2 ratio of methylated : unmethylated transcripts) (13). Mouse models of m.1555A>G do not currently exist owing to technical limitations in mutagenesis of mtDNA, but a transgenic mouse model overexpressing TFB1M was reported to have hearing loss compared with its wild-type counterpart (23,24).

Genetic evidence also supported the involvement of TFB1M in m.1555A>G hearing loss. Non-parametric analysis of families with m.1555A>G and non-syndromic hearing loss showed linkage to markers within the *TFB1M* gene on chromosome 6 (25). Bykhovskaya *et al.* speculated that the m.1555A>G mutation may be more penetrant in the presence of 12S m^6^_2_A rRNA *hypo*methylation, and protective alleles in TFB1M may lead to 12S m^6^_2_A rRNA *hyper*methylation (25).

However, in the present study, we have shown that all 12S rRNA transcripts appear to be m^6^_2_A-methylated in primary RNA in both patients and controls. This contrasts with previous observations in m.1555A>G 143B.TK− osteosarcoma cell cybrids in which there is a 1 : 2 ratio of methylated : unmethylated transcripts in control and a 1 : 1 ratio of methylated : unmethylated transcripts in m.1555A>G RNA (13). Our evidence from primary and non-primary cells suggests that in *H. sapiens* large quantities of unmethylated 12S rRNA are unlikely to exist in the normal situation. The unmethylated 12S rRNA found in non-primary cells may be an artefact of tissue culture, passage number or the aberrant cancerous nuclear genome - known to have altered DNA methylation (26,27). DNA methylation has been shown to differ between blood lymphocytes and lymphoblastoid cells lines, and methylation patterns seen in cell lines may not always be representative of the methylation present in the patient lymphocytes (27). There is considerable variability in relation to levels of m^6^_2_A methylation in the literature; however, our evidence in primary *H. sapiens* cellular RNA is consistent with independent lines of evidence in bacteria and higher plants in which all detectable 12S rRNA appears to be fully methylated (23,28).

Previous work had suggested that m.1583A 12S m^6^_2_A rRNA methylation is detectable in 143B.TK− cells. As we have also shown that it is not possible to detect m.1583A methylation if m.1584A is m^6^_2_A-methylated, our data indicate that previous observations may be an artefact of the radioactive primer extension method used in previous work (13). Our data also strongly suggest that m.1584A is m^6^_2_A methylated by TFB1M before m.1583A in *H. sapiens*, which is in agreement with the structural evidence suggesting that the bacterial homologue of TFB1M, KsgA, methylates the bacterial equivalent of m.1584A prior to m.1583A (29).

Clinically, the concept of mRNA misreading is consistent with the unique sensitivity of patients with m.1555A>G to aminoglycoside antibiotic-associated hearing loss, which is believed to occur when the fidelity of translation of proteins required for OXPHOS is insufficient to sustain the basic energy requirements of cochlear cells. Misreading increases in the presence of aminoglycosides (11). It is also consistent with three of the four candidate genes reported to modify the penetrance of m.1555A>G, leading to hearing loss in the absence of aminoglycoside exposure. Candidate nuclear modifiers of m.1555A>G include: mitochondrial transcription optimization 1 (*MTO1*); GTP binding protein 3 (*GTPBP3*); and 5-methylaminomethyl-2-thiouridylate methyltransferase (*TRMU*). MTO1, GTPBP3 and TRMU are tRNA modification enzymes, involved in the metabolism of mitochondrial tRNAs (mt-tRNAs), particularly the post-transcriptional modification at position 34 on five specific mt-tRNAs (Fig. 1B) (6–8). Modification at mt-tRNA position 34 modulates the ability of the mitochondrial ribosome to discriminate mRNA codons with a discriminator, wobble base (e.g. mt-tRNAs for leucine and phenylalanine) (Fig. 1B). Variants in these candidate modifier genes are believed to increase levels of mRNA misreading in patients with non-syndromic m.1555A>G hearing loss (6–8).

In light of our evidence, the concept of mRNA misreading demonstrated by Hobbie *et al*. stands as the remaining mechanistic hypothesis for both non-syndromic hearing loss and aminoglycoside ototoxicity in patients with m.1555A>G (11). Therefore, strategies to reduce the binding affinity of aminoglycoside antibiotics to *H. sapiens* mitochondrial ribosomes, to increase the threshold accuracy level of m.1555A>G ribosomes and/or to mitigate the mitochondrial dysfunction triggered by aminoglycosides represent the greatest opportunity to shape the development of future patient interventions (30–32).

Twenty years from the discovery of m.1555A>G, the mechanism of action of this mutation and particularly factors determining its penetrance will be of interest to the wider mitochondrial disease community. Thus, continued study of factors influencing the penetrance of homoplasmic mtDNA mutations such as m.1555A>G, without the confounding contribution of heteroplasmy, may unlock our understanding of nuclear modifier genes governing the tissue specificity and clinical variability associated with many severe mitochondrial diseases (33).

Given the large difference that appears to exist between the cybrid m.1555A>G RNA and primary patient m.1555A>G RNA, we propose that RNA methylation studies in transformed cells should be validated in primary clinical samples to ensure that they are applicable to the human situation. RNA epigenetics is a growing area of disease biology, and validation in primary RNA samples is essential to maximise clinical relevance of RNA epigenetics (34,35).

## CLINICAL INFORMATION

Family 1 consists of two siblings and their parents (Fig. 5). Neither child has been exposed to aminoglycosides; both were born without perinatal problems, and neither had been in hospital prior to cochlear implantation. In the first child, parents became concerned about hearing at 1 year of age and severe sensorineural hearing loss (SNHL) was diagnosed around 3 years. By 9 years of age, his hearing loss had progressed to profound and he underwent bilateral cochlear implantation aged 18.5 years, following implantation of his sister (see below). There was no obvious family history of hearing loss, but audiometry revealed that both mother and maternal grandmother had mild high-frequency SNHL.

Case III-2 is the younger sister of case III-1. She was born 3.5 years later following a normal pregnancy and delivery. Her hearing was tested by auditory brainstem responses (ABR) and otoacoustic emissions (OAE) at 6 weeks: ABR showed normal click-evoked responses, and OAE were clearly demonstrable on the left, less so on the right. At age 2 years, she was re-tested and found to have a moderate-to-severe hearing loss. Hearing loss was profound in the left ear by 3.5 years and in the right ear by 5.5 years. She received a unilateral cochlear implant aged 10 years with a second implant at 15 years. Clinical examination of both siblings was normal. She was diagnosed with m.1555A>G aged 4 years, at the same time as her brother, who was then aged 7 years.

In Family 2, case II-1 is the first child of unrelated Sudanese parents and was born in the UK at 42 weeks of gestation following a normal pregnancy and uneventful vaginal delivery. She failed newborn hearing screening and was diagnosed with bilateral moderate to severe SNHL by ABR aged 2 months. At 10 months, she had a pyrexial illness with lymphadenopathy and was subsequently diagnosed with autoimmune neutropaenia. She received six doses of co-amoxiclav intravenously, but no aminoglycosides. She was diagnosed with m.1555A>G aged 3 years 8 months and underwent bilateral cochlear implantation at this age because her hearing loss was profound. She has never received aminoglycosides.

Case II-2 is her younger brother. Delivery was normal but, in view of prolonged rupture of membranes (PROM), he was given five doses of benzyl penicillin (147 mg) and two doses of gentamicin (14.7 mg), the first at birth and the second 24 h later according to standard protocols for PROM. He failed newborn hearing screening aged 3 weeks, and hearing loss was confirmed at 4 weeks. ABR testing at 3 months showed bilateral profound hearing loss. He was tested and found to have m.1555A>G at 13 months and received bilateral cochlear implants aged 2 years but has had no other hospital admissions. Clinical examination was normal in both siblings. Both parents have normal hearing up to and including 8 kHz, but mother has a maternal history of hearing loss starting in the fourth decade (her maternal grandmother, one maternal aunt and two maternal uncles).

Family 3 consists of twins, their older brother and their mother who are Ghanaian in origin. The first child was born following a normal pregnancy and delivery. In the twin pregnancy, there were no complications before 36 weeks when there was PROM. Both twins were delivered by Caesarian section because of a maternal history of uterine fibroids requiring myomectomy. Twin 1 was a female weighing 3.01 kg, and Twin 2 (the proband), a male weighing 2.89 kg. In view of PROM, both twins were given Benzyl Penicillin and Gentamicin according to protocol for PROM. They received two doses, levels were normal and medication was stopped on Day 3. Both twins passed the newborn hearing screen.

Twin 2 had normal speech development, but he was found to have a hearing loss on school entry at 4 years. Current testing at age 8 years shows him to have a moderate asymmetrical hearing loss, worse in the high frequencies [maximum 60 decibels hearing level (dBHL) at 2 kHz in the right ear and 50 dbHL at 4 kHz in the left ear]. Following discovery of hearing loss in Twin 2, Twin 1 who had expressive language delay at age 7 years was tested; she has a mild–moderate high-frequency hearing loss, milder than her brother. The 20-year-old brother of the proband has normal hearing. The mother has a mild bilateral hearing loss, which reaches moderate in the high frequencies. In the family history, there is also reported childhood-onset hearing loss in the maternal uncle and aunt (mother's maternal half-siblings), and adult-onset hearing loss in the maternal grandmother and maternal great uncle.

## METHODS

### Synthetic RNA oligonucleotides

Methylation was detected using a primer extension protocol (Fig. 3). Unmethylated and methylated model RNA oligonucleotides were synthesized (Thermo Scientific) to replicate each of the reported native RNA conditions (12,13). One microliter of 10–20 nm RNA oligonucleotide was incubated with 1 µl of 50–100 nm 5′-Ned-labelled primer and 20 units of RNAse Inhibitor (NEB) at 55°C for 20 min. Twenty units of Avian Myeloblastosis Virus (AMV) Reverse Transcriptase (NEB), ×10 reaction buffer, 20 units of RNase Inhibitor (NEB) and 1 µl 500 nm dNTP stock (containing ddGTP in place of dGTP) were added, and the reaction mixture was incubated overnight at 42°C in a final volume of 10 µl. One microliter was mixed with 8.95 µl Hi-Di Formamide (Applied Biosystems) and 0.05 µl GeneScan Rox size standard (Applied Biosystems) and run on an ABI3130 Genetic Analyser (Applied Biosystems). GeneMarker software (SoftGenetics) was used for fragment analysis. Reactions were performed in triplicate.

The 38-bp, HPLC-purified, 5′ Ned-labelled DNA primer used was 5′ ACATCATCATCATCATCATCATTCGGTTCGTCCAAGTG 3′. A 25-bp tail sequence was added to make the primer 38 bp in length, the minimum reliable length that is compatible with fragment separation on the ABI3130 sequencer. The synthetic, HPLC-purified, model RNA oligonucleotides used (Dharmacon) were as follows: 5′ AAGUGUACUGGAAAGUGCACUUGGACGAAC 3′ for the unmethylated RNA template, 5′ CUGGAdmAAGUGCACUUGGACGAACC 3′ for the template methylated at the equivalent of m.1584A, 5′ CUGGdmAAAGUGCACUUGGACGAACC 3′ for the template methylated at the equivalent of m.1583A and 5′ CUGGdmAdmAAGUGCACUUGGACGAACC 3′ for the template methylated at the equivalent of m.1584A and m.1583A. dm in the oligonucleotide sequence represents m^6^_2_A (N6, N6-dimethyladenosine).

### Patient tissues and cell lines

Ethical approval for this work was obtained from the National Research Ethics Service Committee London Bloomsbury, UK. Total RNA was isolated from 12 control and 14 m.1555A>G patient lymphocyte samples using a Tempus RNA Isolation Kit (Applied Biosystems) and concentrated using 3 K centrifugal units (Millipore). m.1555A>G was detected using restriction digestion and Sanger sequencing. First, a 339-bp fragment was amplified by PCR (forward primer 5′ GCTCAGCCTATATACCGCCATCTTCAGCAA 3′ and reverse primer 5′ TTTCCAGTACACTTACCATGTTACGACTGG 3′) (3). Samples were digested using *Hae*III restriction digestion according to the manufacturer’s instructions and fragments resolved on a 2% w/v TBE agarose gel (3). Digestion of the wild-type allele results in two fragments 216 bp and 123 bp in length. The m.1555A>G mutation creates a novel *Hae*III restriction site, resulting in three fragments 216, 93 and 30 bp in length (3). All samples showing a restriction site gain were sequenced by Sanger sequencing to confirm the presence of the m.1555A>G mutation.

Control and m.1555A>G primary fibroblast cells were cultured in high-glucose Dulbecco's Modified Eagle Medium (DMEM) (Gibco) supplemented with 10% foetal bovine serum (FBS) (Sigma) and 50 µg/ml uridine (Sigma). Fibroblasts were isolated using an established isolation protocol in the absence of aminoglycoside antibiotics. Control and m.1555A>G lymphoblastoid cells were cultured in high-glucose RPMI (Life Technologies) supplemented with 10% FBS (Sigma). The cancerous bone 143B.TK- and 143B.TK-ρ^0^ 206 cell lines were a kind gift from Dr J.-W. Taanman. The HeLa and 143B.TK− cell lines were cultured in high-glucose DMEM supplemented with 10% FBS. The 143B.TK− ρ^0^ cell line was supplemented with 50 μg/ml uridine. Cells were free of mycoplasma infection (Promocell).

### Primer extension—native RNA

Total RNA (10 µg) was incubated with 4 nm 5′-Ned-labelled primer at 55°C for 20 min. Twenty units of AMV Reverse Transcriptase (NEB), ×10 reaction buffer, 20 units of RNase Inhibitor (NEB) and 1 µl of 500 nm dNTP stock (containing ddGTP in place of dGTP) were added. The reaction was incubated overnight at 42°C, desalted using 3K-centrifugal filters (Millipore), vacuum desiccated and re-suspended in 8.95 µl HiDi Formamide (Applied Biosystems), 1 µl dH_2_O and 0.05 µl GeneScan Rox size standard (Applied Biosystems). Reactions were run and analysed as described previously. Samples were analysed in triplicate.

## SUPPLEMENTARY MATERIAL

Supplementary Material is available at *HMG* online.

## FUNDING

This work was supported by UCL Grand Challenges, Sparks Children's Charity, Great Ormond Street Hospital Children's Charity and the NIHR Biomedical Research Centre at Great Ormond Street Hospital for Children NHS Trust. Funding to pay the Open Access publication charges for this article was provided by University College London.

## Supplementary Material

Supplementary Data
