# Supplementary material for: Mitochondrial m.1584A 12S m62A rRNA methylation in families with m.1555A>G associated hearing loss
Source: Hum Mol Genet. 2014 Oct 9;24(4):1036–44. doi: 10.1093/hmg/ddu518 (PMC4986548; doi:10.1093/hmg/ddu518)
Supplement: Supplementary Data [file supp_24_4_1036__index.html]

Mitochondrial m.1584A 12S m62A rRNA methylation in families with m.1555A>G associated hearing loss — Mitochondrial m.1584A 12S m62A rRNA methylation in families with m.1555A>G associated hearing loss — Mitochondrial m.1584A 12S m62A rRNA methylation in families with m.1555A>G associated hearing loss — Supplementary Data 

# Mitochondrial m.1584A 12S m62A rRNA methylation in families with m.1555A>G associated hearing loss

## Supplementary Data

Supplementary Data

**Files in this Data Supplement:**

- Supplementary Data - Docx file
- Supplementary Figure 1 - tif file
- Supplementary Figure 2 - tif file
- Supplementary Figure 3 - tif file
