# Supplementary material for: Mitochondrial m.1584A 12S m62A rRNA methylation in families with m.1555A>G associated hearing loss
Source: Hum Mol Genet. 2014 Oct 9;24(4):1036–44. doi: 10.1093/hmg/ddu518 (PMC4986548; doi:10.1093/hmg/ddu518)
Supplement: Supplementary Data [file supp_ddu518_ddu518supp.docx]

**Supplementary Figure Legends**

**S. Figure 1** RNA electropherograms of the 14 patients analysed in this study. Patients were analysed in triplicate with the exception of four patients where there was insufficient RNA. These patients were analysed in duplicate as indicated.

S. Figure 2 Primer extension analysis of a 143B.TK- osteosarcoma cell line, and a 143B.TK- ρ^0^ cell line lacking mtDNA. (A) The 143B.TK- cell line is partially methylated at m.1584A and partially unmethylated. (B) The 143B.TK- ρ^0^ cell line does not contain any mtDNA or 12S; lack of primer extension on RNA isolated from this cell line confirms specificity of the primer to the mitochondrial transcriptome.

**S. Figure 3** Synthetic methylated and unmethylated RNA oligonucleotides, sequence replicates of the native 12S rRNA, were mixed together in the same primer extension reaction. Increasing concentrations of unmethylated RNA template could be detected by the assay in a dose dependent manner. A ratio of methylated: unmethylated template of 1: 0.22 was the lowest ratio detected.
