# Supplementary figures and images for: Mitochondrial m.1584A 12S m62A rRNA methylation in families with m.1555A>G associated hearing loss
Source: Hum Mol Genet. 2014 Oct 9;24(4):1036–44. doi: 10.1093/hmg/ddu518 (PMC4986548; doi:10.1093/hmg/ddu518)

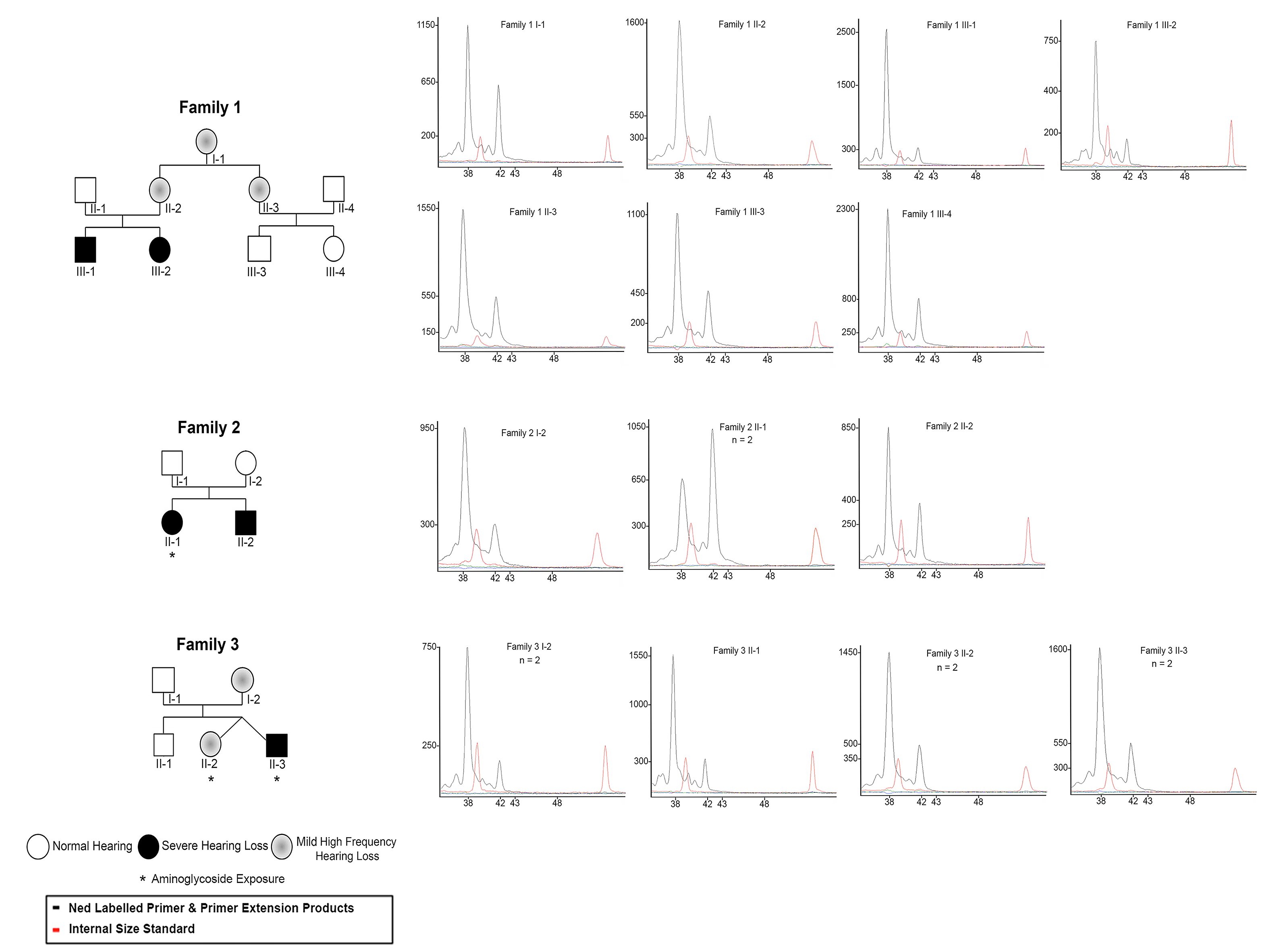

Supplement: Supplementary Data [file supp_ddu518_ddu518supp_fig1.tif]

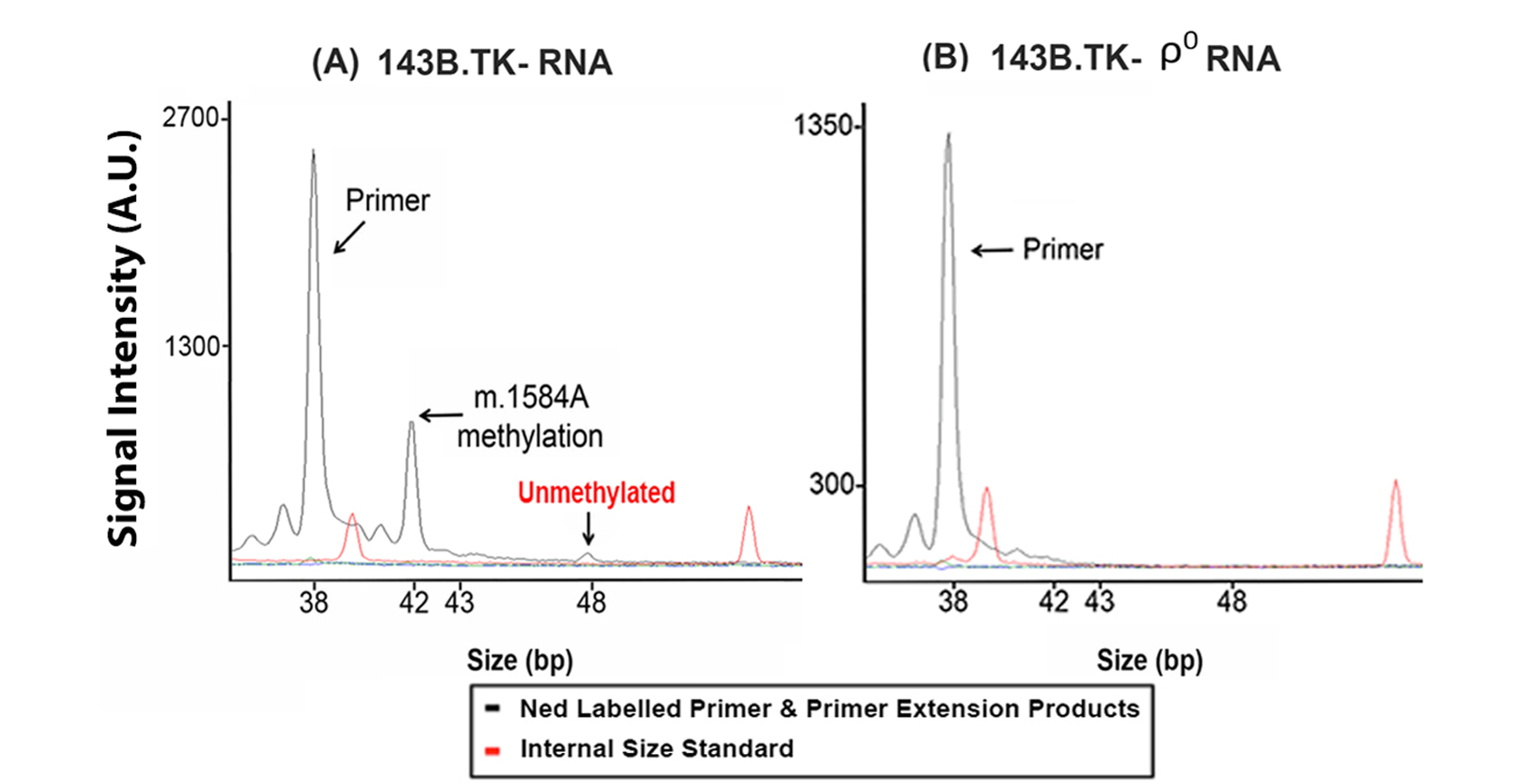

Supplement: Supplementary Data [file supp_ddu518_ddu518supp_fig2.tif]

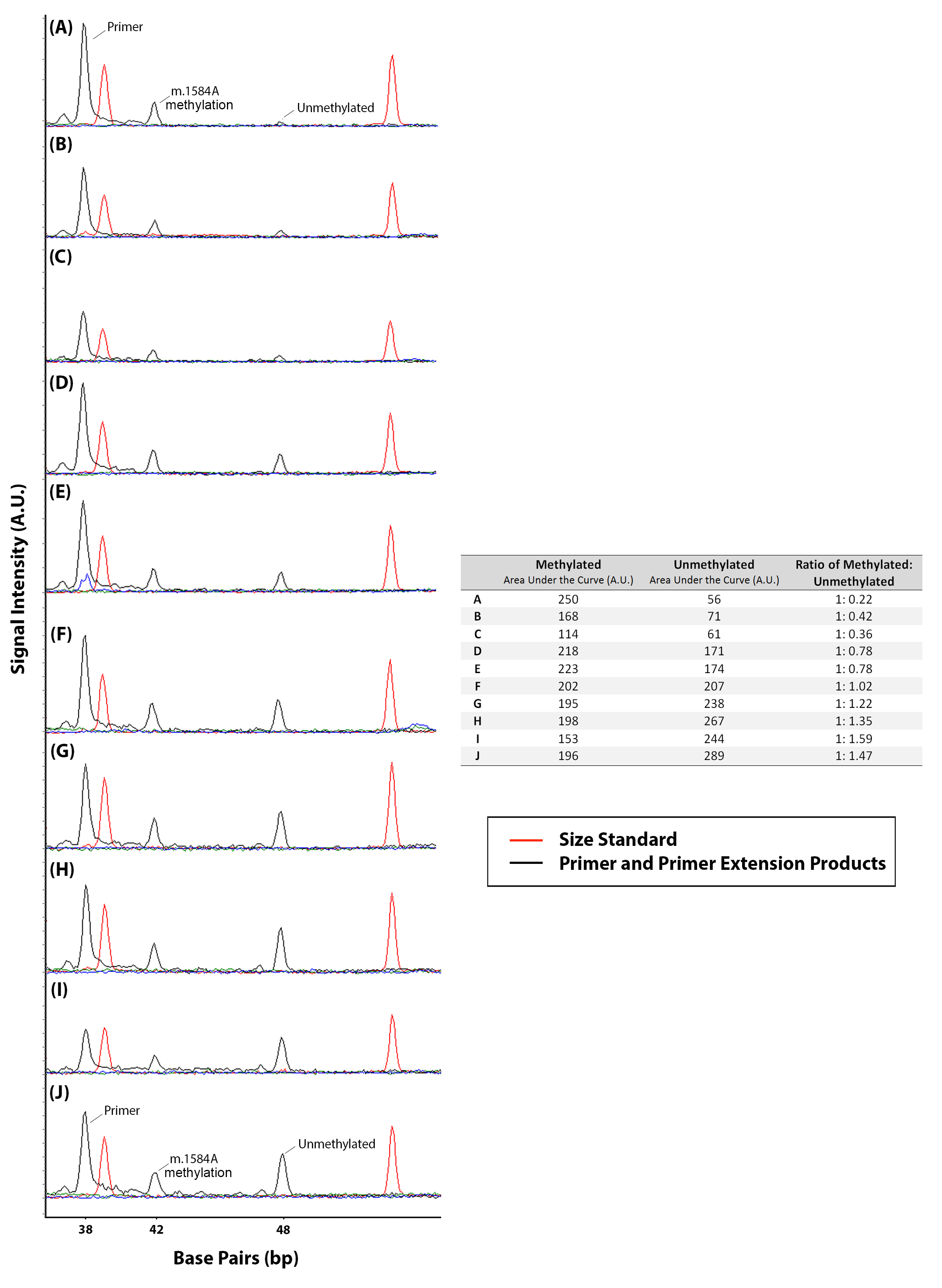

Supplement: Supplementary Data [file supp_ddu518_ddu518supp_fig3.tif]
